# Deterministic neural networks as sources of uncorrelated noise for probabilistic computations

**DOI:** 10.1186/1471-2202-16-S1-P62

**Published:** 2015-12-18

**Authors:** Jakob Jordan, Tom Tetzlaff, Mihai Petrovici, Oliver Breitwieser, Ilja Bytschok, Johannes Bill, Johannes Schemmel, Karlheinz Meier, Markus Diesmann

**Affiliations:** 1 Institute for Neuroscience and Medicine (INM-6) and Institute for Advanced Simulation (IAS-6), Jülich Research Center and JARA, Jülich, Germany; 2 Kirchhoff Institute for Physics, Ruprecht-Karls-University Heidelberg, Heidelberg, Germany; 3 Institute for Theoretical Computer Science, Graz University of Technology, Graz, Austria

## 

Neural-network models of brain function often rely on the presence of noise [1-4]. To date, the interplay of microscopic noise sources and network function is only poorly understood. In computer simulations and in neuromorphic hardware [5-7], the number of noise sources (random-number generators) is limited. In consequence, neurons in large functional network models have to share noise sources and are therefore correlated. In general, it is unclear how shared-noise correlations affect the performance of functional network models. Further, there is so far no solution to the problem of how a limited number of noise sources can supply a large number of functional units with uncorrelated noise.

Here, we investigate the performance of neural Boltzmann machines [2-4]. We show that correlations in the background activity are detrimental to the sampling performance and that the deviations from the target distribution scale inversely with the number of noise sources. Further, we show that this problem can be overcome by replacing the finite ensemble of independent noise sources by a recurrent neural network with the same number of units. As shown recently, inhibitory feedback, abundant in biological neural networks, serves as a powerful decorrelation mechanism [8,9]: Shared-noise correlations are actively suppressed by the network dynamics. By exploiting this effect, the network performance is significantly improved. Hence, recurrent neural networks can serve as natural finite-size noise sources for functional neural networks, both in biological and in synthetic neuromorphic substrates. Finally we investigate the impact of sampling network parameters on its ability to faithfully represent a given well-defined distribution. We show that sampling networks with sufficiently strong negative feedback can intrinsically suppress correlations in the background activity, and thereby improve their performance substantially.
